# Control group changes in objectively measured physical activity in primary care: protocol for a systematic review and meta-analysis

**DOI:** 10.1186/s13643-019-1060-2

**Published:** 2019-06-18

**Authors:** Nicole Freene, Rachel Davey, Steven M. McPhail

**Affiliations:** 1 Physiotherapy, Faculty of Health, University of Canberra, Bruce, 2617 Australia; 2 Health Research Institute, University of Canberra, Bruce, 2617 Australia; 3 Centre for Research & Action in Public Health, University of Canberra, Bruce, 2617 Australia; 4 School of Public Health & Social Work and Institute of Health and Biomedical Innovation, Queensland University of Technology, Victoria Park Road, Kelvin Grove, 4059 Australia; 5 Centre for Functioning and Health Research, Metro South Health, Corner of Ipswich Road and Cornwall Street, Buranda, 4103 Australia

**Keywords:** Control, Accelerometry, Pedometer, Measurement, Measurement reactivity, Question-behaviour effect

## Abstract

**Background:**

There is some evidence that simply measuring physical activity alone can increase self-reported physical activity behaviour. Objective measures of physical activity in intervention studies have increased substantially over the last decade. Yet, there is no synthesised evidence of observed changes in the control group physical activity in trials that have used objective physical activity measurement approaches. Understanding factors associated with control group increases (or decreases) in physical activity may have implications for planning physical activity research and in clinical settings where objective measures of physical activity may be used. The aim of this systematic review is to describe changes in objectively measured physical activity that have occurred within control groups in primary care physical activity intervention studies and, if possible, identify factors that are potentially associated with these changes.

**Methods:**

The PRISMA-P reporting guidelines for systematic review protocols will be followed. Five electronic databases (PubMed, MEDLINE, SPORTDiscuss, PsychINFO, CINAHL) will be searched to identify physical activity controlled (randomised, cluster, quasi-experimental) studies conducted with adults in primary care. Search terms will be based on previous systematic reviews, and only peer-reviewed articles published in English will be considered. The main outcome measure is the change in objectively measured physical activity within the control group. Risk of bias will be assessed using the Cochrane Collaboration tool and the Risk Of Bias in Non-randomised Studies—of Interventions tool. Meta-analyses will be conducted where possible among studies with sufficient homogeneity.

**Discussion:**

This systematic review and meta-analysis will determine the extent to which physical activity measurement alone is associated with changes in objectively measured physical activity levels in control groups in primary care. Findings from this study will inform future physical activity intervention research and practice. If measuring physical activity alone is associated with increases in physical activity levels that may be considered beneficial for health, this could indicate that measurement alone may be a low cost, efficient and effective method to increase a proprotion of the population’s physical activity levels.

**Systematic review registration:**

PROSPERO CRD42018104896

## Background

Physical inactivity remains a global health challenge. Half of all adults in most developed countries are not meeting the public health physical activity guidelines [1]. Sufficient physical activity reduces the risk of premature death from all causes and several chronic diseases, such as cardiovascular disease, diabetes mellitus and some cancers [2]. Numerous physical activity interventions have been investigated to encourage increased levels of physical activity in adults, with positive results unlikely to be sustained over the longer term or on a large scale [3]. Brief physical activity interventions that are effective, low cost and easily implemented into real-world practice have a wide reach and are able to be sustained over a long time period are needed. ‘Measurement as intervention’ may be a suitable approach.

Measurement reactivity, and the mere-measurement effect, or the question-behaviour effect, have been reported in the physical activity literature [4, 5]. Measurement reactivity is defined as being present where the act of measurement leads to changes in the people being measured [4]. The mere-measurement effect, or the question-behaviour effect, refers to the change in behaviour under investigation following measurement of the behaviour and/or related cognitions [5]. These mechanisms are recognised as potential challenges when calculating sample sizes and interpreting results of controlled physical activity intervention studies due to the likely small increase in physical activity levels in the control group, making it difficult to interpret measurement and intervention effects [4, 5]. To determine how often this may occur in primary care, Waters et al. conducted a systematic review finding that approximately one third of physical activity intervention studies in primary care have reported improvements in self-reported physical activity among participants who were in the control group [6].

The Solomon four-group design has been utilised in limited studies to separate intervention and measurement effects [7]. In a large randomised controlled study in Dutch general practice (*n* = 635), different frequencies of physical activity measurement were compared using a Solomon four-group design [8]. Participants were randomised to three or one physical activity measurements over 6 months. More participants in the three physical activity measurement groups met the physical activity guidelines at the end of the intervention period as compared to the one physical activity measurement group when considering self-reported physical activity, but there was no difference found between groups for a subsample of participants using accelerometry. The authors concluded that the increased frequency of physical activity measurements affected the participants’ self-reported physical activity behaviour. These findings suggest that completing questionnaires about physical activity may not affect objectively assessed physical activity, with more evidence for measurement affecting self-reports of behaviour than objectively measured behaviour [4], although there have been some reports of measurement reactivity using accelerometers [9, 10] and pedometers [11].

Waters et al. and Opdenacker et al. also found that control group changes in physical activity were more likely when follow-up assessments were carried out over a longer period of time [6, 12]. Waters et al. found in their systematic review that follow-up assessments completed at 9 months as compared to 7 months were more likely to result in a clinically meaningful improvement in physical activity in control groups. Opdenacker et al. found that at 2 years, with a 12-month no intervention follow-up, there was no difference in aerobic fitness between the two intervention groups (structured vs lifestyle) and a control group, concluding this was consistent with the control group improvement in physical activity and this was possibly due to a measurement effect.

In primary care, systematic reviews and reviews of reviews have found that physical activity interventions have a small to moderate positive effect [13–15], although the most effective physical activity elements, including intervention intensity and participant characteristics, in this setting are unclear [13, 14]. Considering physical activity maintenance interventions, Murray suggested that primary care may not be the most appropriate setting for promoting maintenance in healthy populations, finding limited effectiveness in this population in primary care [3]. It is important to note that the large majority of the studies included in these reviews used self-report measures. In view of the possible implications of measurement reactivity, or the mere-measurement effect, this may account for some of the small effects.

There is evidence of control group improvements in physical activity, to a similar level as intervention groups in some cases, particularly over the longer term [6]. This may indicate that with minimal contact and resources, physical activity behaviour change may be achievable. However, synthesis of this evidence to date has focused on self-reported physical activity. The objective measurement of physical activity (e.g. accelerometers, pedometers) is considered a superior method of physical activity measurement compared with self-report measures, with lower levels of variability observed for validity and reliability, despite some pragmatic limitations [16]. This has resulted in a significant increase in objective physical activity measures used within intervention studies in the last 10 years, increasing from 4% of lifestyle physical activity intervention studies in 2006 to 71% in 2016 [17]. Currently, there appears to be no research investigating the observed changes in the control group physical activity in trials that have used objective physical activity measurement approaches. The aim of this systematic review is to estimate the effect of control group participation and measurement on objectively measured physical activity in adults. The systematic review will answer the following research questions:

1. How much does objectively measured physical activity increase (or decrease) within control groups in primary care physical activity intervention trials?
2. What factors are associated with control group changes in objectively measured physical activity, considering potential factors such as length of trial, frequency of measurement, intensity of physical activity measurements, and participant characteristics?

## Methods

### Design

To facilitate the design and reporting of this protocol for a systematic review and meta-analysis, the Preferred Reporting Items for Systematic Reviews and Meta-Analyses Protocol (PRISMA-P) statement will be followed [18]. In addition, the systematic review has been guided by the systematic review completed by Waters et al. investigating control group improvements in self-reported physical activity in primary care [6]. In accordance with the guidelines, our systematic review protocol was registered with the International Prospective Register of Systematic Reviews (PROSPERO) on 12 September 2018 (registration number CRD42018104896).

### Eligibility criteria

#### Types of studies

Randomised controlled trials (RCTs), cluster RCTs and quasi-experimental (controlled) studies that aimed to increase physical activity will be included. Consistent with the systematic review completed by Waters et al., studies using multiple behaviour interventions will be included if a component of the intervention specifically aimed to increase physical activity levels [6]. Studies will be excluded if there was no control or comparison group (e.g. compared two or more physical activity interventions, or no comparison group). All other study types (e.g. cohort studies, cross-sectional studies) will be excluded. Only articles available in English will be included. There will be no restrictions placed on the date of publication.

#### Types of participants

Study participants must be adults (18 years or older). Participants may be healthy, at risk of a chronic disease, or have been diagnosed with a chronic disease (e.g. type 2 diabetes, coronary heart disease, chronic obstructive pulmonary disease) where participants may have been included in the trial based on this diagnosis.

#### Setting

All physical activity intervention studies need to be completed in primary care. A primary care setting was selected due to its broad reach and the number of physical activity intervention studies completed in this setting. Primary health care is defined as the first point of contact people have with the health system [19, 20]. It provides community-based care, meeting the health needs of individuals throughout life [19]. Primary care includes general practice, allied health services, community health and community pharmacy [20]. Physical activity interventions conducted in the workplace, university and all other settings will be excluded.

#### Types of outcome measures

##### Objectively measured physical activity

The primary outcome measure is the change in objectively measured physical activity (e.g. accelerometer, pedometer) within the control group. Doubly labelled water and indirect calorimetry will not be included in this systematic review due to the limited feasibility of these measures in a primary care setting [16]. Objectively measured physical activity may be a primary or secondary outcome measure within physical activity intervention trials. Studies where the change in control group objectively measured physical activity between baseline and immediately post-intervention is not reported, or not able to be calculated, will be excluded. The main metrics for objectively measured physical activity considered will be minutes per day or week of moderate-to-vigorous physical activity (MVPA), proportion of participants meeting the minimum public health recommendations for MVPA (150 min of MVPA per week) [21], and step counts. All units or methods of objectively measured MVPA will be included.

Additional data will be extracted when considering the objectively measured physical activity. This includes mode of administration, duration between assessments, number of interim assessments, time between interim assessments and intensity of physical activity measurements [6]. Mode of administration of the objective measurement tool will be coded as interviewer-administered or self-administered (e.g. accelerometer/pedometer delivered via post to participants). Assessment duration will be the length of time between baseline and immediately post-intervention measurements, the main end point. If the trial includes additional follow-up objective measurements of physical activity, this data will also be extracted. The number of interim assessments will be recorded as the number of times the physical activity was objectively measured in addition to baseline and post-intervention measurements. The time between interim assessments will be recorded. Measurement intensity will be calculated as the total number of physical activity assessments administered to each participant at a single data collection point. Physical activity assessments may include any additional objective measures (e.g. pedometer, activity tracker), subjective measures (e.g. questionnaire, diary), objective measures of cardiovascular fitness (e.g. functional fitness tests, maximum oxygen consumption testing, heart rate monitoring), measurement of other constructs related to physical activity (e.g. stage of change, self-efficacy), and measurement of other health behaviours (e.g. diet, alcohol consumption). Measurement intensity will be recorded as a maximum score of 6, with 1 point for the primary objective physical activity measure, and 1 point for each additional measure as listed above [6].

##### Participant characteristics

Sex, mean age, body mass index and health status at baseline will be recorded for each control group. Health status will be coded as healthy, at risk of a chronic disease, or have been diagnosed with a chronic disease (e.g. type 2 diabetes, coronary heart disease, chronic obstructive pulmonary disease).

##### Control group intervention

Control group intervention will be coded as no additional contact beyond measurement (true control), an alternative intervention unrelated to physical activity (attention control), usual care only, usual care plus print information addressing physical activity, or usual care plus physical activity tailored advice delivered via personal feedback or verbally (< 5 min consultation) [6].

#### Search strategy

The literature search strategy is based on the systematic review by Waters et al. and has been further developed by the authors from examining existing literature and systematic reviews on objective measurement of physical activity [6, 16]. One author (NF) will search PubMed and MEDLINE, SPORTDiscus, PsycINFO, and CINAHL via the Ebsco interface. The electronic databases will be searched for the terms (Exercise OR “physical activity” OR “physical fitness” OR “motor activity”) AND (“Primary health care” OR “physicians, family” OR “general practi*” OR “primary care” OR “family practi*”) AND (Intervention OR “intervention studies”) AND (Objective* OR acceleromet* OR “activity monitor” OR “motion sensor” OR pedom* OR “Heart Rate Monitor*” OR “Direct Observation”). No study design or date limits will be imposed on the search. Where able, the searches will be limited to articles published in peer-reviewed journals, English language, human participants and adults. The reference lists of all studies selected for the review will be examined to identify further studies, as well as those of previous reviews.

#### Selection process

Articles will be imported into Rayyan, a free web and mobile application for systematic reviews, and all duplicates will be removed [22]. Two reviewers (NF, SM) will independently screen titles and abstracts of all articles to identify potentially relevant papers. Full texts of potentially relevant articles will be obtained and independently reviewed by two reviewers (NF, SM) for inclusion. Reasons for excluding trials will be recorded. Disagreements between reviewers at any stage of screening will be resolved by consensus or by a third reviewer (RD). The reviewers will not be blinded to journal titles or study authors. A PRISMA flow diagram will be used to record the number of studies included and excluded at each stage of the selection process.

#### Data collection process

A data extraction form will be created and completed by two reviewers independently (NF, SM). Data extracted will include demographic information (publication details), methodology (study design, sample size, statistical analysis) and all outcome measures. Means, or medians, and measures of dispersion for the control group objectively measured physical activity at baseline, immediately post-intervention and, if relevant, follow-up will be recorded. Mean changes in the control group objectively measured physical activity will be recorded and if, not reported, calculated by subtracting the mean value at baseline from the mean value immediately post-intervention and follow-up [6]. The primary outcome measure is the change in the control group objectively measured physical activity from baseline to immediately post-intervention. Within-control group statistically significant changes and effect sizes will be recorded or calculated, where possible. Differences in means or medians of ≥ 10% will be considered noteworthy [6]. Results from an intention-to-treat analysis will be used, when possible. For trials that use more than one objective measure of physical activity (e.g. MVPA mins/day, steps/day), both measures will be recorded. In cases where more than one publication reports the same data source, only one article per data source will be retained to avoid double counting. If study authors use an objective measure of physical activity (e.g. accelerometers, pedometer) but do not report on these outcomes in the manuscript, the authors will be contacted to determine whether the objectively measured physical activity data can be obtained. Disagreements between reviewers will be resolved by consensus or by a third reviewer (RD). Study authors will be contacted to resolve any other uncertainties, with a maximum of two contact attempts made to obtain additional information.

#### Risk of bias

To assess the possible risk of bias, the Cochrane Collaboration tool for assessing risk of bias will be used for randomised and cluster randomised controlled studies, and the Risk Of Bias in Non-randomised Studies—of Interventions (ROBINS-I) tool will be used for quasi-experimental studies. The Cochrane risk of bias tool covers sequence generation, allocation concealment, blinding, incomplete outcome data and selective outcome reporting [23]. The ROBINS-I tool covers bias due to confounding, selection of participants into the study, classification of interventions, deviations from intended interventions, missing data, measurement of outcomes and selection of the reported result [24]. All studies will be assessed for risk of bias by two reviewers independently, with any conflicting results reviewed by a third reviewer. Study authors will be contacted for more information if needed. Risk of bias for each randomised and cluster randomised study will be categorised as low, unclear or high risk [23]. Risk of bias for quasi-experimental studies will be classified as no information, low, moderate, serious or critical risk [24].

#### Analysis

A narrative synthesis of the literature will be completed. This will include the characteristics of included studies and summary of effects reported within each study, as well as comparing and contrasting changes in physical activity levels between studies on the basis of study (and sample) characteristics (including physical activity assessment methodology). Where possible, outcomes will be reported on the original scale. If studies are sufficiently homogenous (e.g. design, participants and outcomes), data will be pooled across studies. Physical activity outcomes will be grouped and analysed separately on the basis of data types and study design, where relevant. If meta-analyses are to be conducted and outcomes from different scales are to be combined for this purpose, these outcomes will be standardised as guided by the Cochrane Handbook for Systematic Reviews of Intervention [25]. A random effects model to incorporate heterogeneity among studies is planned. This will include the use of a random effect model(s) and, where relevant, to visualise effect estimates. Forest plots will be created using RevMan 5.3 [26] to synthesise the measures of effect and 95% confidence intervals for the control group changes in objectively measured physical activity, although evidence from dissimilar study designs and outcome types will not be reported in the same forest plot. Heterogeneity will be assessed using the *I*^2^ statistic and interpreted according to the Cochrane guidelines [25]. Publication bias will be examined using a funnel plot if a sufficient number of studies are available for inclusion [27]. Analysis of subgroups will be performed if sufficient data is available. These subgroup analyses may be conducted on the basis of differences between health status, control group intervention, mode of administration, duration between assessments, number of interim assessments, time between interim assessments and intensity of physical activity measurements.

## Discussion

To our knowledge, this systematic review will be the first to examine the efficacy of control group physical activity measurement on objectively measured physical activity levels in primary care. This review will systematically retrieve and examine controlled physical activity intervention studies in primary care, assess risk of bias and synthesise the data using meta-analyses. Findings from this study will inform future physical activity intervention research and practice. If physical activity measurement alone is found to improve objectively measured physical activity levels, it may be a low cost, efficient and effective method to increase a proportion of the populations’ physical activity levels, leading to improved health throughout adulthood and into older age.

## Data Availability

The datasets used and/or analysed during the current study will be available from the corresponding author on reasonable request.

## Abbreviations

MVPA: Moderate-to-vigorous physical activity
PRISMA-P: Preferred Reporting Items for Systematic Reviews and Meta-Analyses Protocol
PROSPERO: International Prospective Register of Systematic Reviews
RCTs: Randomised controlled trials
ROBINS-I: Risk Of Bias in Non-randomised Studies—of Interventions
